# Bicycle crashes and sickness absence - a population-based Swedish register study of all individuals of working ages

**DOI:** 10.1186/s12889-019-7284-1

**Published:** 2019-07-15

**Authors:** Linnea Kjeldgård, Maria Ohlin, Rasmus Elrud, Helena Stigson, Kristina Alexanderson, Emilie Friberg

**Affiliations:** 1 Division of Insurance Medicine, Department of Clinical Neuroscience, Karolinska Institutet, SE-171 77 Stockholm, Sweden; 2 Department of Food and Nutrition and Sport Science, University of Gothenburg, Gothenburg, Sweden; 3 Department of Mechanics and Maritime Sciences, Chalmers University of Technology, Gothenburg, Sweden; 4 Swedish National Road and Transport Research Institute, VTI, P.O Box 8072, 402 78 Gothenburg, SE Sweden; 5 Vehicle Safety Division, Department of Applied Mechanics, Chalmers University of Technology, Gothenburg, Sweden; 6 Folksam Research, Stockholm, Sweden

**Keywords:** Sick-leave, Disability pension, Bicycle crash, Traffic injury, Population-based, Cross-sectional

## Abstract

**Background:**

In recent years, bicycle injuries have increased, yet little is known about the impact of such injures on sickness absence (SA) and disability pension (DP). The aim was to explore SA and DP among individuals of working ages injured in a bicycle crash.

**Method:**

A nationwide register-based study, including all individuals aged 16–64 years and living in Sweden, who in 2010 had in- or specialized out-patient healthcare (including emergency units) after a bicycle crash. Information on age, sex, sociodemographics, SA, DP, crash type, injury type, and injured body region was used. We analyzed individuals with no SA or DP, with ongoing SA or full-time DP already at the time of the crash, and with new SA > 14 days in connection to the crash. Crude and adjusted odds ratios (OR) with 95% confidence intervals for new SA were estimated by logistic regression.

**Results:**

In total, 7643 individuals had healthcare due to a new bicycle crash (of which 85% were single-bicycle crashes). Among all, 10% were already on SA or full-time DP at the time of the crash, while 18% had a new SA spell. The most common types of injuries were external injuries (38%) and fractures (37%). The body region most frequently injured was the upper extremities (43%). Women had higher OR (1.40; 1.23–1.58) for new SA than men, as did older individuals compared with younger (OR 2.50; 2.02–3.09, for ages: 55–64 vs. 25–34). The injury types with the highest ORs for new SA, compared with the reference group external injuries was fractures (8.04; 6.62–9.77) and internal injuries (7.34; 3.67–14.66). Individuals with traumatic brain injury and injuries to the vertebral column and spinal cord had higher ORs for SA compared with other head, face, and neck injuries (2.72; 1.19–6.22 and 3.53; 2.24–5.55, respectively).

**Conclusions:**

In this explorative nationwide study of new bicycle crashes among individuals of working ages, 18% had a new SA spell in connection to the crash while 10% were already on SA or DP. The ORs for new SA were higher among women, older individuals, and among individuals with a fracture.

## Introduction

According to Swedish nationwide statistics, bicyclists have in recent years become the road-user group with the highest number of severe injuries [1]. Since bicycling has increased in urban areas in recent years, different stakeholders have given more attention to create a safer road environment for bicyclists. This is especially the case in cities, where bicycling is an important complement to reduce vehicle congestion and greenhouse gas emissions [2]. Bicycling is also important as a way of increasing physical activity in the population. Several recent studies have highlighted the positive health impacts of increased bicycling [3–5]. Nevertheless, bicycling also involves some risks for the bicyclists [2, 6], e.g., a recent study observed 29 times higher risk for injury among bicyclists compared with car occupants [7]. A majority of those injuries are nonfatal but could lead to long-term consequences, hence, focus on nonfatal outcomes is essential [3]. Long-term sickness absence (SA) or disability pension (DP) among injured individuals are consequences of road traffic accidents that impact the individual as well as the family, colleagues, employer, insurers, and society [8, 9].

So far, there is only very limited scientific knowledge about SA and DP after a bicycle crash. Sickness absence has been shown to be a relatively common outcome after a road traffic injury [10–12], however, to the best of our knowledge, only four studies on bicyclists and risk of SA have been published [12–15]. Three of these studies are more than 20 years old and based on relatively small samples. Furthermore, those three studies have not taken into consideration if the individuals already were on SA or DP at the time of the crash, nor sociodemographic factors of the injured individuals, such as educational level, country of birth, or marital status [12–14], that is, factors that in previous studies and reviews were shown to impact risks of SA/DP [9, 16–19]. The fourth is a recent large study where we investigated duration of SA after a bicycle crash [15]. Only individuals with no or new SA in connection to the crash were included in that study. In this study also individuals with ongoing SA or fulltime DP were included as well as factors such as type of injury. To get a broad understanding of both SA and DP in connection to a bicycle crash, the aim of the present study was to explore SA and DP among all individuals of working ages who were injured in a bicycle crash, both in general and by different sociodemographic factors, crash type, type of injury, and injured body region.

## Materials and methods

A population-based register study was conducted. The study population included all individuals aged 16–64 years, living in Sweden 31 December 2009, who in 2010 received in- or specialized out-patient healthcare (including at emergency units) due to an injury from a new bicycle crash. We had no information about primary healthcare (e.g., visits to general practitioner/family doctors).

Data from five nationwide registers from the following three authorities were used and linked at individual level, using the unique personal identity number assigned to all residents in Sweden:

- From *Statistics Sweden*, the “Longitudinal integration database for health insurance and labour market studies” (LISA) was used for identifying all 16–64 years old individuals living in Sweden 31 December 2009, *N* = 5 982 221 and for sociodemographic information (sex, age, educational level, country of birth, type of living area, and marital status).

- From the *National Board of Health and Welfare,* the in- and specialized out-patient register was used to identify the study population and for medical information related to the injury. The cause of death register was used to identify which of those individuals who had died in the first 30 days after the injury.

- From the *Swedish Social Insurance Agency*, the register, “Micro-data for analyses of the social insurance” (MiDAS) was used for dates and grades of SA and DP.

**Fig. 1:**
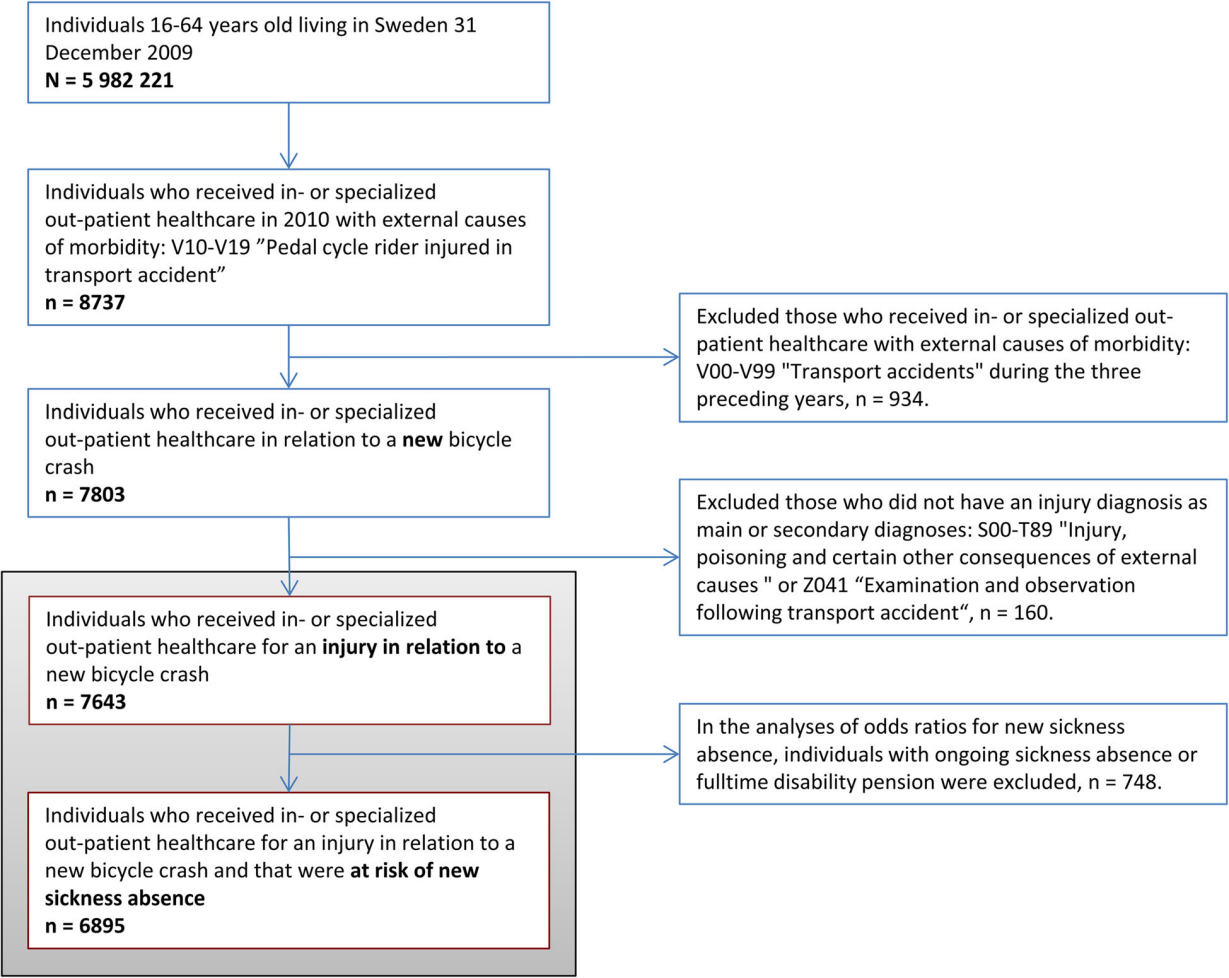
Flowchart of study population, inclusion criteria, and exclusion criteria

In the national patient register, that holds information on all in- and specialized out-patient healthcare, including emergency visits, both diagnoses (one main and all secondary diagnoses) and external causes of morbidity are recorded according to the International Statistical Classification of Diseases and Related Health Problems; ICD-10 [20]. Individuals having received in- or specialized out-patient healthcare in 2010 due to bicycle crashes were identified by codes for external causes of morbidity V10-V19: “Pedal cycle rider injured in transport accident” (*n* = 8737). Since the actual date of the crash is not known to us, the date of the in- or specialized out-patient healthcare visit/hospitalization is here after referred to as the *crash date*. In order to include only new bicycle crashes, the individuals who during the three years prior to their crash date received in- or specialized out-patient healthcare for a bicycle or another transport-related injury (ICD10 external causes of morbidity V00-V99: “Transport accidents”) were excluded (*n* = 934), leaving 7803 individuals. Furthermore, those who did not have an injury diagnosis as main or secondary diagnoses (ICD10: S00-T89 “Injury, poisoning and certain other consequences of external causes” or Z04.1 “Examination and observation following transport accident”) were excluded, leaving a study population of 7643 individuals (Fig. 1).

Out of the 7643 individuals, nine individuals died within 30 days after the bicycle crash.

Based on type of crash, the individuals were categorized into the following three groups: single-bicycle crash (V17, V18, V19.3, V19.8, V19.9) (reference group); collision with pedestrian, animal, or other bicycle (V10, V11); and collision with motor vehicle (V12-V16, V19, V19.0, V19.1-V19.2, V19.4-V19.6). Type of healthcare was also categorized into three groups as: only specialized out-patient healthcare (reference group); in-patient healthcare ≤1 day; and in-patient healthcare > 1 day. The cut-off for in-patient healthcare was based on the median duration of hospital stay among those receiving such healthcare.

According to the patient register, some individuals had up to three different healthcare visits registered on the crash date. Each such visit had a main diagnosis, determined as such by the treating physician, and could also have a number of additional secondary diagnoses. For categorization purposes, we selected one injury diagnosis per individual, in the following way: The main injury diagnosis was selected over secondary injury diagnoses, the diagnoses for in-patient healthcare over out-patient healthcare, and injury before other types of diagnoses (S00-S99 over T00-T88, T00-T88 over Z04.1). The majority (78%) had only one injury diagnosis while 15% had two. A modified version of the Barell matrix [21] was used to classify the ICD-10 codes into categories of type of injury and injured body region, a similar categorization was used in a recent study on car occupants [11]. Thus, type of injury was categorized into the following six groups: fracture; dislocation; sprains and strains; internal (brain, spinal cord, and other internal organs); external (open wounds, contusions and superficial injuries) (reference group); and “other and unspecified”. The injured body region was categorized into eight groups: traumatic brain injury (not concussion); concussion; other head, face and neck (reference group); vertebral column and spinal cord; torso; upper extremities; lower extremities; and “other and unspecified”.

The sociodemographic covariates were categorized as: sex (women; men (reference group)), age group (16–24; 25–34 (reference group); 35–44; 45–54; 55–64 years), level of education (elementary (≤9 years); high school (9–12 years); university/college (> 12 years) (reference group)), country of birth (Sweden (reference group); not Sweden), type of living area (big cities (reference group); medium-sized cities; small cities/villages), marital status (married (reference group); not married). Reference groups were chosen based on size of the groups and expected proportions with new SA, with larger groups or groups expected to have lower proportions of new SA being used as the reference.

All individuals living in Sweden, ≥16 years old, and with income from work, unemployment, or parental-leave benefits can apply for SA benefits from the Social Insurance Agency if having a disease or injury that leads to reduced work capacity [22]. The first day of a SA spell is an unreimbursed qualifying day (more days for self-employed). A physician’s certificate is required after day 7. For most employees, day 2–14 are reimbursed by the employer [22]. For others, e.g., unemployed, the Social Insurance Agency administrates benefits from the first day of SA, thus information also on shorter SA spells was available for these individuals. In order not to introduce a bias, only information on SA spells > 14 days was used. All individuals aged 19–64 can be granted DP if disease or injury leads to long-term or permanent work incapacity. Both SA and DP can be granted for full- or part-time (100, 75, 50, 25%) of ordinary work hours. That is, someone on part-time DP can at the same time have part-time SA. For the calculation of mean and median net days of SA (for SA > 14 days) SA was summed to whole days (e.g. two days of 50% part-time SA was counted as 1 net day). Benefit for SA amount up to 80% of lost income up to a certain level, for DP 64% of lost income up to a certain level.

Individuals were categorized into three groups regarding SA/DP situation at the time of the crash as follows: already ongoing SA/DP; new SA; and no new SA. To be defined as already having ongoing SA/DP, the SA spell had to have started at least five days before the crash date and still be ongoing at the crash date. When assessing SA, any SA spells regardless of grade were included. Considering DP, only full-time DP was categorized as already being on DP. A new SA spell in relation to the bicycle crash was defined as a SA spells that had started someday between 4 days before and 4 days after the crash date and that lasted for > 14 days. To handle the fact that individuals might not have received in- or specialized out-patient healthcare the first days after the actual crash date, e.g., sought primary healthcare first, or not applied for SA benefits the first days, due to e.g., holidays, a spread of starting days for SA in relation to the crash date was allowed. The timespan of ±4 days was chosen based on distribution of start dates of SA in relation to crash date. Further, the group “no new SA” includes not only individuals without any SA or DP but also individuals with ongoing part-time DP but no new SA spell > 14 days.

### Statistical analyses

The individual’s sociodemographics (sex, age, level of education, country of birth, type of living area, and marital status), crash type, type and duration of healthcare, type of injury, and injured body region were shown by SA and DP status at the time for the bicycle crash, using descriptive statistics.

Odds ratios (OR) and 95% confidence intervals (CI) for new SA were estimated by logistic regression. In these analyses, individuals with already ongoing SA or full-time DP were excluded (*n* = 748) – that is, individuals not at risk of new SA, leaving 6895 individuals. First, the OR for new SA by the sociodemographic factors were calculated, in univariate models (crude), then, mutually adjusted (model 1), as well as adjusted by the crash and injury characteristics (model 2). These analyses were also stratified by sex. Then, the OR for new SA for the characteristics of the crash were estimated, first adjusted for sociodemographic factors (model 1) and then for crash type, type of injury, and injured body region (model 2 and 3), and in model 4 and 5 both the sociodemographic factors and the crash and injury characteristics were taken into consideration. Also, sensitivity analyses excluding individuals on part-time DP were performed.

The statistical analyses were performed using SPSS (version 22) and STATA (version 14).

## Results

In total, 7643 individuals of working ages received in- or specialized out-patient healthcare due to a new bicycle crash in 2010 (Table 1). In the study population, there were a somewhat higher proportion of men (57%), while the proportions of individuals in each age group were similar. High proportions of individuals were born in Sweden (85%), lived in medium-sized cities (42%), were not married (68%), and had high school or college/university education (77%).

**Table 1:** Study-population^1^ sociodemographics, by sickness absence (SA) and disability pension (DP) status at bicycle crash date

	All		Ongoing SA/DP		New SA		No new SA	
	*n*	column%	*n*	row%	*n*	row%	*n*	row%
Total	7643	100	748	9.8	1367	17.9	5528	72.3
Sex								
Women	3303	43.2	332	10.1	678	20.5	2293	69.4
Men	4340	56.8	416	9.6	689	15.9	3235	74.5
Age group, years								
16–24	1576	20.6	41	2.6	74	4.7	1461	92.7
25–34	1217	15.9	80	6.6	167	13.7	970	79.7
35–44	1580	20.7	121	7.7	323	20.4	1136	71.9
45–54	1746	22.8	213	12.2	400	22.9	1133	64.9
55–64	1524	19.9	293	19.2	403	26.4	828	54.3
Level of education								
Elementary	1748	22.9	245	14.0	200	11.4	1303	74.5
High school	3260	42.7	368	11.3	702	21.5	2190	67.2
University/College	2635	34.5	135	5.1	465	17.6	2035	77.2
Country of birth								
Sweden	6481	84.8	624	9.6	1175	18.1	4682	72.2
Not Sweden	1162	15.2	124	10.7	192	16.5	846	72.8
Type of living area								
Big cities	2584	33.8	239	9.2	415	16.1	1930	74.7
Medium-sized cities	3195	41.8	293	9.2	560	17.5	2342	73.3
Small cities/villages	1864	24.4	216	11.6	392	21.0	1256	67.4
Marital status								
Married	2442	32.0	203	8.3	589	24.1	1650	67.6
Not married	5201	68.0	545	10.5	778	15.0	3878	74.6

^1^All individuals in Sweden of working ages who in 2010 had a new bicycle crash leading to in- or specialized out-patient healthcare

Most of the individuals (72%) did not have any ongoing SA spell > 14 days or full-time DP at the time of the crash, nor a new SA spell. In total, 1367 individuals (18%) had new SA and 748 individuals (10%) were already on SA or full-time DP at the time of the crash. Among all with ongoing DP, 72.5% were on full-time DP. Having a new SA spell was more common among older individuals and among women. Among those with new SA, the median number of net days of the new SA spells was 33 days, the mean was 61 days. Most of the individuals with a new SA spell had full-time SA (92.6%).

Most individuals, 6484 (85%), were injured in a single-bicycle crash and most (83%) had only specialized out-patient healthcare (Table 2). Among those with in-patient healthcare > 1 day, the proportion with a new SA spell was high (51%) when compared with those hospitalized ≤1 day (24%), or compared with those with only specialized out-patient healthcare (14%). External injuries and fractures were the most common injury types, accounting for 39 and 37% of all injuries, respectively. The most commonly injured body regions were the upper extremities (43%) followed by the lower extremities (19%) and other head, face, and neck not including traumatic brain injuries (19%). New SA was most common among individuals with a fracture (33%) and among individuals with injuries to the vertebral column and spinal cord (37%).

**Table 2:** Crash and injury characteristics, by sickness-absence (SA) and disability-pension (DP) status at bicycle crash date^1^

	All		Ongoing SA/DP		New SA		No new SA	
	*N*	column%	*n*	row%	*n*	row%	*n*	row%
Total	7643	100	748	9.8	1367	17.9	5528	72.3
Crash type								
Single	6484	84.8	641	9.9	1146	17.7	4697	72.4
Collision with pedestrian, animal, or other bicycle	431	5.6	35	8.1	82	19.0	314	72.9
Collision with motor vehicle	728	9.5	72	9.9	139	19.1	517	71.0
Healthcare								
Only specialized out-patient healthcare	6345	83.0	578	9.1	879	13.9	4888	77.0
In-patient healthcare ≤1 day	643	8.4	70	10.9	153	23.8	420	65.3
In-patient healthcare > 1 day	655	8.6	100	15.3	335	51.1	220	33.6
Type of injury								
Fracture	2805	36.7	340	12.1	930	33.2	1535	54.7
Dislocation	326	4.3	17	5.2	79	24.2	230	70.6
Sprains and strains	679	8.9	70	10.3	80	11.8	529	77.9
Internal	733	9.6	82	11.2	103	14.1	548	74.8
External	2942	38.5	230	7.8	161	5.5	2551	86.7
Other and unspecified	158	2.1	9	5.7	14	8.9	135	85.4
Body region								
Traumatic Brain Injury, not concussion	117	1.5	24	20.5	42	35.9	51	43.6
Concussion	563	7.4	51	9.1	46	8.2	466	82.8
Other head, face and neck	1433	18.7	98	6.8	93	6.5	1242	86.7
Vertebral column and spinal cord	156	2.0	12	7.7	58	37.2	86	55.1
Torso	546	7.1	64	11.7	89	16.3	393	72.0
Upper extremities	3264	42.7	308	9.4	733	22.5	2223	68.1
Lower extremities	1452	19.0	182	12.5	304	20.9	966	66.5
Other and unspecified	112	1.5	9	8.0	2	1.8	101	90.2

^1^Among all individuals in Sweden of working ages who in 2010 had a new bicycle crash leading to in- or specialized out-patient healthcare

In the analysis of OR of new SA, only those 6895 individuals, at risk were included. The adjusted OR for a new SA among women compared with men was 1.55 (95% CI 1.34–1.78) (Table 3). The OR for new SA was higher among older individuals. For individuals with high-school education compared with those with university/college education, the OR was 1.77 (95% CI 1.52–2.07). When stratifying by sex and after adjusting for potential confounders, results remained similar for both women and men. Type of living area was associated with new SA among men (OR 1.28; 95% CI 1.05–1.58 for medium-sized cities and OR 1.57; 95% CI 1.25–1.97 for small cities/villages; both compared with big cities), but not among women (OR 0.89; 95% CI 0.72–1.11 for medium-sized cities and OR 1.15; 95% CI 0.91–1.47 for small cities/villages; both compared with big cities).

**Table 3:** Odds ratios (OR) for new sickness absence (SA) following a bicycle crash, by sociodemographics

	*N*^1^ (%SA)	Crude	Model 1	Model 2
		OR (95% CI^2^)	OR (95% CI)	OR (95% CI)
Sex				
Women	2971 (22.8)	1.39 (1.23–1.56)	1.40 (1.23–1.58)	1.55 (1.34–1.78)
Men	3924 (17.6)	ref.		
Age group, years				
16–24	1535 (4.8)	0.29 (0.22–0.39)	0.27 (0.20–0.36)	0.27 (0.20–0.38)
25–34	1137 (14.7)	ref.	ref.	ref.
35–44	1459 (22.1)	1.65 (1.34–2.03)	1.52 (1.23–1.88)	1.54 (1.22–1.94)
45–54	1533 (26.1)	2.05 (1.68–2.50)	1.85 (1.51–2.28)	1.67 (1.33–2.10)
55–64	1231 (32.7)	2.83 (2.31–3.46)	2.50 (2.02–3.09)	1.85 (1.46–2.34)
Level of education				
Elementary	1503 (13.3)	0.67 (0.56–0.80)	1.14 (0.93–1.39)	1.20 (0.96–1.50)
High school	2892 (24.3)	1.40 (1.23–1.60)	1.56 (1.36–1.79)	1.77 (1.52–2.07)
University/College	2500 (18.6)	ref.	ref.	ref.
Country of birth				
Sweden	5857 (20.1)	ref.	ref.	ref.
Not Sweden	1038 (18.5)	0.90 (0.76–1.07)	0.91 (0.77–1.09)	0.93 (0.76–1.14)
Type of living area				
Big cities	2345 (17.7)	ref.	ref.	ref.
Medium-sized cities	2902 (19.3)	1.11 (0.97–1.28)	1.08 (0.93–1.25)	1.20 (1.02–1.42)
Small cities/villages	1648 (23.8)	1.45 (1.24–1.70)	1.36 (1.16–1.61)	1.41 (1.17–1.70)
Marital status				
Married	2239 (26.3)	ref.	ref.	ref.
Not Married	4656 (16.7)	0.56 (0.50–0.63)	0.91 (0.79–1.04)	0.91 (0.78–1.05)

^1^*N* = 6895, i.e., excluding those already on SA or full-time disability pension, among all individuals in Sweden of working ages who in 2010 had a new bicycle crash leading to in- or specialized out-patient healthcare

^2^CI Confidence intervals

Model 1 adjusted for: Age, Sex, Level of education, Country of birth, Type of living area, Marital status

Model 2 adjusted for: factors as in model 1 as well as, Crash type, Type of injury, Body region

Regarding the crash and injury characteristics, having had in-patient healthcare > 1 day was strongly associated with new SA both regarding crude OR 8.47 (95% CI 7.04–10.18) and after adjusting for sociodemographic factors and for crash- and injury-related factors (OR 7.54 (95% CI 6.20–9.17)) (Table 4). Also, the type of crash was, in the fully adjusted model, associated with new SA, high OR was observed for collision with motor vehicle compared with single bicycle crashes. When examining the type of injury, the category containing fractures had 8 times higher adjusted OR for SA compared with external injuries. When analysing OR for new SA regarding body region, the category: vertebral column and spinal cord had a high OR compared with the category of other head, face and neck, as did lower extremities and traumatic brain injury (not concussion).

**Table 4:** Odds ratios for new sickness absence following a bicycle crash, by crash and injury characteristics^1, 2^

			Sociodemographics	Crash and injury characteristics		Crash, injury, and sociodemographic characteristics	
		Crude	Model 1	Model 2	Model 3	Model 4	Model 5
All at risk of SA	*n* (% SA)	OR (95% CI)	OR (95% CI)	OR (95% CI)	OR (95% CI)	OR (95% CI)	OR (95% CI)
Crash type							
Single	5843 (19.6)	ref.	ref.		ref.		ref.
Collision with pedestrian, animal, or other bicycle	396 (20.7)	1.07 (0.83–1.38)	1.06 (0.81–1.37)		1.14 (0.86–1.50)		1.13 (0.85–1.51)
Collision with motor vehicle	656 (21.2)	1.10 (0.90–1.34)	1.11 (0.90–1.36)		1.33 (1.07–1.67)		1.37 (1.08–1.73)
Healthcare							
Only specialized out-patient healthcare	5767 (15.2)	ref.	ref.	ref.		ref.	
In-patient healthcare ≤1 day	573 (26.7)	2.03 (1.66–2.47)	2.05 (1.66–2.52)	2.04 (1.67–2.49)		2.06 (1.67–2.54)	
In-patient healthcare > 1 day	555 (60.4)	8.47 (7.04–10.18)	7.51 (6.18–9.13)	8.51 (7.07–10.24)		7.54 (6.20–9.17)	
Type of injury							
Fracture	2465 (37.7)	9.60 (8.03–11.48)	9.48 (7.88–11.4)	9.85 (8.23–11.79)	8.27 (6.85–9.99)	9.74 (8.09–11.73)	8.04 (6.62–9.77)
Dislocation	309 (25.6)	5.44 (4.03–7.35)	5.24 (3.83–7.16)	5.70 (4.21–7.71)	4.77 (3.48–6.54)	5.48 (4.00–7.49)	4.36 (3.15–6.05)
Sprains and strains	609 (13.1)	2.40 (1.80–3.18)	2.55 (1.90–3.41)	2.46 (1.85–3.27)	1.64 (1.22–2.20)	2.62 (1.96–3.51)	1.77 (1.31–2.40)
Internal	651 (15.8)	2.98 (2.29–3.88)	3.01 (2.29–3.94)	3.01 (2.31–3.92)	7.28 (3.72–14.22)	3.04 (2.32–3.99)	7.34 (3.67–14.66)
External	2712 (5.9)	ref.	ref.	ref.	ref.	ref.	ref.
Other and unspecified	149 (9.4)	1.64 (0.93–2.91)	1.66 (0.93–2.98)	1.57 (0.88–2.79)	2.84 (1.54–5.24)	1.62 (0.90–2.90)	2.83 (1.51–5.31)
Body region							
Traumatic brain injury, not concussion	93 (45.2)	11.00 (6.95–17.41)	9.47 (5.86–15.3)	10.95 (6.91–17.34)	3.19 (1.43–7.09)	9.40 (5.81–15.20)	2.72 (1.19–6.22)
Concussion	512 (9.0)	1.32 (0.91–1.91)	1.36 (0.93–1.97)	1.32 (0.91–1.91)	0.39 (0.18–0.83)	1.36 (0.93–1.98)	0.38 (0.18–0.83)
vOther head, face and neck	1335 (7.0)	ref.	ref.	ref.	ref.	ref.	ref.
Vertebral column and spinal cord	144 (40.3)	9.01 (6.07–13.36)	9.20 (6.09–13.91)	8.89 (5.98–13.22)	3.65 (2.37–5.63)	9.06 (5.98–13.72)	3.53 (2.24–5.55)
Torso	482 (18.5)	3.02 (2.21–4.13)	2.67 (1.94–3.67)	3.00 (2.20–4.11)	1.73 (1.23–2.45)	2.66 (1.93–3.66)	1.48 (1.04–2.11)
Upper extremities	2956 (24.8)	4.40 (3.51–5.52)	4.45 (3.53–5.62)	4.42 (3.53–5.55)	2.07 (1.61–2.65)	4.48 (3.55–5.65)	2.09 (1.61–2.70)
Lower extremities	1270 (23.9)	4.20 (3.28–5.38)	4.05 (3.14–5.22)	4.20 (3.28–5.38)	3.13 (2.39–4.09)	4.05 (3.14–5.22)	2.81 (2.12–3.72)
Other and unspecified	103 (1.9)	0.26 (0.06–1.09)	0.29 (0.07–1.19)	0.26 (0.06–1.08)	0.29 (0.07–1.28)	0.29 (0.07–1.18)	0.29 (0.07–1.29)

^1^*N* = 6895, i.e. excluding those already on SA or full-time disability pension, among all individuals in Sweden of working ages who in 2010 had a new bicycle crash leading to in- or specialized out-patient healthcare

^2^Abbreviations: *SA* Sickness absence, *OR* Odds ratios, *CI* Confidence intervals

Model 1, adjusted for: Age, Sex, Level of education, Country of birth, Type of living area, Marital status

Model 2, adjusted for: Crash type

Model 3, adjusted for: Crash type, Type of injury, Body region

Model 4, adjusted for: factors as in model 1 as well as, Crash type

Model 5, adjusted for: factors as in model 4 as well as, Type of injury, Body region

Excluding individuals with part-time DP did not change the results (data not shown).

## Discussion

In this nationwide study in Sweden, investigating SA among all individuals of working age who in 2010 had a new bicycle crash leading to in- or specialized out-patient healthcare, we found that 10% already were on SA or on full-time DP at the crash date and that as many as 18% had a new SA spell lasting > 14 days. This indicates that SA > 14 days is a common consequence of a bicycle crash. Moreover, women, older individuals, and individuals with high school educational level had higher odds for such new SA. Being hospitalized > 1 day compared with only out-patient healthcare, having a fracture compared with external injuries, or a traumatic brain injury (not concussion) compared with injuries to other head, face and neck was associated with higher OR for new SA.

In recent years there has been a focus on creating a safer environment for bicyclists [2]. Despite efforts to reduce the number of road users who are killed or injured, these attempts have not been as successful for bicyclists as for car occupants. Bicyclists have in recent years become the most frequently injured road-user group in Sweden [1]. Except for the recent study we did, investigating duration of SA after a bicycle crash [15], to the best of our knowledge there are only three previous studies on bicycle crashes and SA [12–14]. Those three studies are all old and with small number of patients selected from specific hospitals in Nordic counties, e.g., not covering the whole population [12–14]. In the present study we found that 18% had new SA after a bicycle crash. This is in line with one of those previous studies, including 447 individuals in Sweden with data from 1978 to 79 of which 19% had SA benefits [12]. However, that study included individuals of all ages, also those not of working age, and it is not clear if the individuals already on SA were included in the study nor whether also short-term SA were included or not. Without such information, the results are difficult to compare to our results. We found that among those with in-patient healthcare lasting more than one day, half (51%) had a new SA spell > 14 days. This result is in line with a study in Sweden of 791 patients (where 190 were bicyclist) who after a road traffic accident in 1970 had in-patient healthcare at one general surgery ward; 50% of them had no SA or were on SA for less than one week [13].

A recent study about car occupants with similar inclusion criteria observed lower proportion of new SA spells (10% vs. 18% observed here) [11]. The higher proportion of individuals who had a new SA after a bicycle crash could possibly be explained by the fact that bicyclist as unprotected road users are at higher risk for injuries [7]. This highlights the importance of gaining further knowledge on aspects of bicycle crashes and their consequences as bases for preventive actions.

Differences in injury patterns between single bicycle crashes and bicycle-car crashes have been shown in previous studies [23–25]. Based on hospital data, most of the crashes are single-bicycle crashes (70–77%) e.g. [2, 12, 24, 25]. This is in line with our findings were 85% of the bicycle crashes were single crashes. The slightly higher proportion of single-bicycle crashes is possibly related to the use of both in-patient and specialized out-patient registers in the present study.

We found that upper extremities was the most commonly injured body region (43%) followed by head (when combining concussion, traumatic brain injury and other head, face, and neck injuries) with 28% of the injuries, which is in line with five previous studies showing upper extremities and/or the head to be the most commonly injured body regions [12, 14, 23–25]. However, none of these previous studies have included risk of SA as a consequence of injuries to different body regions. We found external injuries to be the most common type of injury; this was also the injury type with the lowest OR for new SA. The second most common type of injury was fracture (37%). Fractures had ten-fold OR for SA compared with external injuries. Furthermore, in our study we found that individuals with traumatic brain injury, not concussion, had an eleven-fold OR for SA compared with individuals with other head, face and neck injuries. Individuals with a vertebral column and spinal cord injury (standing for just 2 % of the injuries) had a nine-fold OR for SA. Thus, the individuals with the most commonly injured body regions or type of injury did not have the highest ORs for receiving new SA, but contributed with many cases of new SA due to the number of injured individuals (Table 4), this should be considered in further studies and interventions.

Strengths of the present study are that data from high-quality nationwide registers were used with total population coverage of all residents in Sweden of working ages, that all data were register-based (thus, certified by a physician) rather than self-reported, no drop-outs, and the very large study population, allowing for sub-group analyses. This study covers all individuals receiving in- and/or specialized out-patient healthcare, including emergency visits, thus all bicycle crashes severe enough to acquire such medical attention were included. In assessments of the possible negative consequences of bicycling, previous studies have mainly referred to fatalities or police-reported crashes [5, 26]. This will not adequately describe the situation, e.g., in Sweden the police reports only cover around 7% of all bicycle crashes [25]. This under-reporting of the number of crashes to the police has also been shown in other countries [27]. Healthcare data cover a much larger proportion of bicycle crashes and the use of such data is, therefore, a strength when studying individuals injured in bicycle crashes [25, 28].

A limitation is that the selection of only one main injury diagnosis might have led to over- or under-estimation of the impact of different diagnoses. However, the majority had only one injury diagnosis registered. It can also be considered a limitation that individuals with a bicycle injury not requiring healthcare or only requiring primary healthcare were not included; that is, our results will underestimate the total number of bicycle crashes, primarily the milder injuries. In this study we focused on SA in direct connection to the bicycle crash, further research is required to elucidate long-term consequences of bicycle crashes.

## Conclusions

In this nationwide study of bicycle crashes among individuals of working ages, 10% were already on SA/DP at the time of the crash while 18% had a new SA spell > 14 days. The vast majority of injuries were due to single-bicycle crashes. The most often injured body region was upper extremities and the most common type of injuries were fractures and external injuries. The ORs for new SA was higher among women, older individuals, and if the crash resulted in a fracture. The ORs for new SA was also high among those with traumatic brain injuries (not concussion), injuries to the vertebral column and spinal cord, and injuries to the upper and lower extremities compared with the category “other head, face and neck”. Further elucidation of these aspects are warranted given the current increase of bicycle crashes.

## Data Availability

The data cannot be made publically available, according to privacy regulations. According to the General Data Protection Regulation, the Swedish law SFS 2018:218, the Swedish Data Protection Act, the Swedish Ethical Review Act, and the Public Access to Information and Secrecy Act, data can only be made available, after legal review, for researchers who meet the criteria for access to this type of sensitive and confidential data. Readers may contact professor Kristina Alexanderson (kristina.alexanderson@ki.se) regarding the data.

## **Abbreviations**

CI: Confidence interval
DP: Disability pension
OR: Odds ratio
SA: Sickness absence

## References

[1] Analysis of Road Safety Trends 2016 – Management by objectives for road safety work towards the 2020 interim targets. In*.*, vol. 098: Swedish Transport Administration; 2017.

[2] Safer cycling - a common strategy for the period 2014–2020. In*.*: Swedish Transport Administration; 2014.

[3] de Hartog Jeroen Johan, Boogaard Hanna, Nijland Hans, Hoek Gerard (2010). Do the Health Benefits of Cycling Outweigh the Risks?. Environmental Health Perspectives.

[4] Rojas-Rueda D, de Nazelle A, Teixido O, Nieuwenhuijsen MJ (2013). Health impact assessment of increasing public transport and cycling use in Barcelona: a morbidity and burden of disease approach. Prev Med.

[5] Holm Astrid Ledgaard, Glümer Charlotte, Diderichsen Finn (2012). Health Impact Assessment of increased cycling to place of work or education in Copenhagen. BMJ Open.

[6] Peden M: World report on road traffic injury prevention World Health Organisation: Geneva 2004. In*.*; 2004.

[7] Nilsson P, Ohlin M, Stigson H, Strandroth J (2016). Modelling the effect on injuries and fatalities when changing mode of transport from car to bicycle. Accident Anal Prev.

[8] Marmot M, Feeney A, Shipley M, North F, Syme SL (1995). Sickness absence as a measure of health status and functioning: from the UK Whitehall II study. J Epidemiol Community Health.

[9] Alexanderson K, Norlund A (2004). Swedish council on technology assessment in health care (SBU). Chapter 1. Aim, background, key concepts, regulations, and current statistics. Scandinavian journal of public health Supplement.

[10] Berecki-Gisolf J, Collie A, McClure R (2013). Work disability after road traffic injury in a mixed population with and without hospitalisation. Accid Anal Prev.

[11] Elrud R, Stigson H, Ohlin M, Alexanderson K, Kjeldgård L, Friberg E: Sickness Absence among Passenger Car Occupants following a Crash. In: *IRCOBI Conference:* 2017*; Antwerp, Belgium*; 2017.

[12] Bjornstig U, Naslund K (1984). Pedal cycling accidents--mechanisms and consequences. A study from northern Sweden. Acta Chir Scand.

[13] Hansson PG (1976). Sick-leave after road traffic accidents. Scand J Soc Med.

[14] Olkkonen S, Lahdenranta U, Slatis P, Honkanen R (1993). Bicycle accidents often cause disability--an analysis of medical and social consequences of nonfatal bicycle accidents. Scand J Soc Med.

[15] Ohlin M, Kjeldgård L, Elrud R, Stigson H, Alexanderson K, Friberg E (2018). Duration of sickness absence following a bicycle crash, by injury type and injured body region: a nationwide register-based study. J Transp Health.

[16] Jansson C, Mittendorfer-Rutz E, Alexanderson K (2012). Sickness absence because of musculoskeletal diagnoses and risk of all-cause and cause-specific mortality: a nationwide Swedish cohort study. Pain.

[17] Wang M, Alexanderson K, Runeson B, Head J, Melchior M, Perski A, Mittendorfer-Rutz E (2014). Are all-cause and diagnosis-specific sickness absence, and sick-leave duration risk indicators for suicidal behaviour? A nationwide register-based cohort study of 4.9 million inhabitants of Sweden. Occup Environ Med.

[18] Allebeck P, Mastekaasa A (2004). Swedish council on technology assessment in health care (SBU). Chapter 3. Causes of sickness absence: research approaches and explanatory models. Scandinavian journal of public health Supplement.

[19] Allebeck P, Mastekaasa A (2004). Swedish council on technology assessment in health care (SBU). Chapter 5. Risk factors for sick leave - general studies. Scandinavian journal of public health Supplement.

[20] International Statistical Classification of Diseases and Related Health Problems (1993). Tenth revision (ICD-10).

[21] Barell V, Aharonson-Daniel L, Fingerhut LA, Mackenzie EJ, Ziv A, Boyko V, Abargel A, Avitzour M, Heruti R (2002). An introduction to the Barell body region by nature of injury diagnosis matrix. Inj Prev.

[22] Social Insurance in Figures 2016. In*.*: Swedish Social Insurance Agency; 2016.

[23] Juhra C, Wieskotter B, Chu K, Trost L, Weiss U, Messerschmidt M, Malczyk A, Heckwolf M, Raschke M (2012). Bicycle accidents - do we only see the tip of the iceberg? A prospective multi-Centre study in a large German city combining medical and police data. Injury.

[24] Stutts JC, Hunter WW (1999). Motor vehicle and roadway factors in pedestrian and bicyclist injuries: an examination based on emergency department data. Accid Anal Prev.

[25] Rizzi M, Stigson H, Krafft M: Cyclist Injuries Leading to Permanent Medical Impairment in Sweden and the Effect of Bicycle Helmets. In: *IRCOBI Conference:* 2013*;* Gothenburg*,* Sweden; 2013.

[26] Hartog JJ, Boogaard H, Nijland H, Hoek G (2011). Do the health benefits of cycling outweigh the risks?. Cien Saude Colet.

[27] Amoros E, Martin JL, Laumon B (2006). Under-reporting of road crash casualties in France. Accid Anal Prev.

[28] Ludvigsson JF, Andersson E, Ekbom A, Feychting M, Kim JL, Reuterwall C, Heurgren M, Olausson PO (2011). External review and validation of the Swedish national inpatient register. BMC Public Health.

